# Prognostic indices in stereotactic radiotherapy of brain metastases of non-small cell lung cancer

**DOI:** 10.1186/s13014-015-0550-1

**Published:** 2015-11-26

**Authors:** David Kaul, Alexander Angelidis, Volker Budach, Pirus Ghadjar, Markus Kufeld, Harun Badakhshi

**Affiliations:** Department of Radiation Oncology, Charité School of Medicine and University Hospital, Campus Virchow-Klinikum, Augustenburger Platz 1, 13353 Berlin, Germany; Charité CyberKnife Center, Charité School of Medicine and University Hospital, Campus Virchow-Klinikum, Augustenburger Platz 1, 13353 Berlin, Germany

**Keywords:** Brain metastases, Radiation therapy, Survival, Prognostic scores

## Abstract

**Background:**

Our purpose was to analyze the long-term clinical outcome and to identify prognostic factors after Linac-based stereotactic radiosurgery (SRS) or fractionated stereotactic radiotherapy (FSRT) on patients with brain metastases (BM) from non-small cell lung cancer (NSCLC).

**Materials and Methods:**

We performed a retrospective analysis of survival on 90 patients who underwent SRS or FSRT of intracranial NSCLC metastases between 04/2004 and 05/2014 that had not undergone prior surgery or whole brain radiotherapy (WBRT) for BM. Follow-up data was analyzed until May 2015. Potential prognostic factors were examined in univariable and multivariable analyses. The Golden Grading System (GGS), the disease-specific graded prognostic assessment (DS-GPA), the RADES II prognostic index as well as the NSCLC-specific index proposed by Rades et al. in 2013 (NSCLC-RADES) were calculated and their predictive values were tested in univariable analysis.

**Results:**

The median follow-up time of the surviving patients was 14 months.

The overall survival (OS) rate was 51 % after 6 months and 29.9 % after 12 months.

Statistically significant factors of better OS after univariable analysis were lower International Union Against Cancer (UICC) stage at first diagnosis, histology of adenocarcinoma, prior surgery of the primary tumor and lower total BM volume. After multivariable analysis adenocarcinoma histology remained a significant factor; higher Karnofsky Performance Score (KPS) and the presence of extracranial metastases (ECM) were also significant.

The RADES II and the NSCLC-RADES indices were significant predictors of OS. However, the NSCLC-RADES failed to differentiate between intermediate- and low-risk patients. The DS-GPA and GGS were not statistically significant predictors of survival in univariable analysis.

**Conclusion:**

The ideal prognostic index has not been defined yet. We believe that more specific indices will be developed in the future. Our results indicate that the histologic subtype of NSCLC could add to the prognostic value of specialized future indices. The RADES II index had the highest predictive value in the examined patient cohort.

## Background

Brain metastases (BM) are four to five times more common than primary intracranial malignancies [1] and 20–40 % of cancer patients will develop such lesions in the course of their disease [2]. Non-small cell lung cancer (NSCLC) is one of the leading causes of BM accounting for 18–64 % of all lesions [3].

**Fig. 1:**
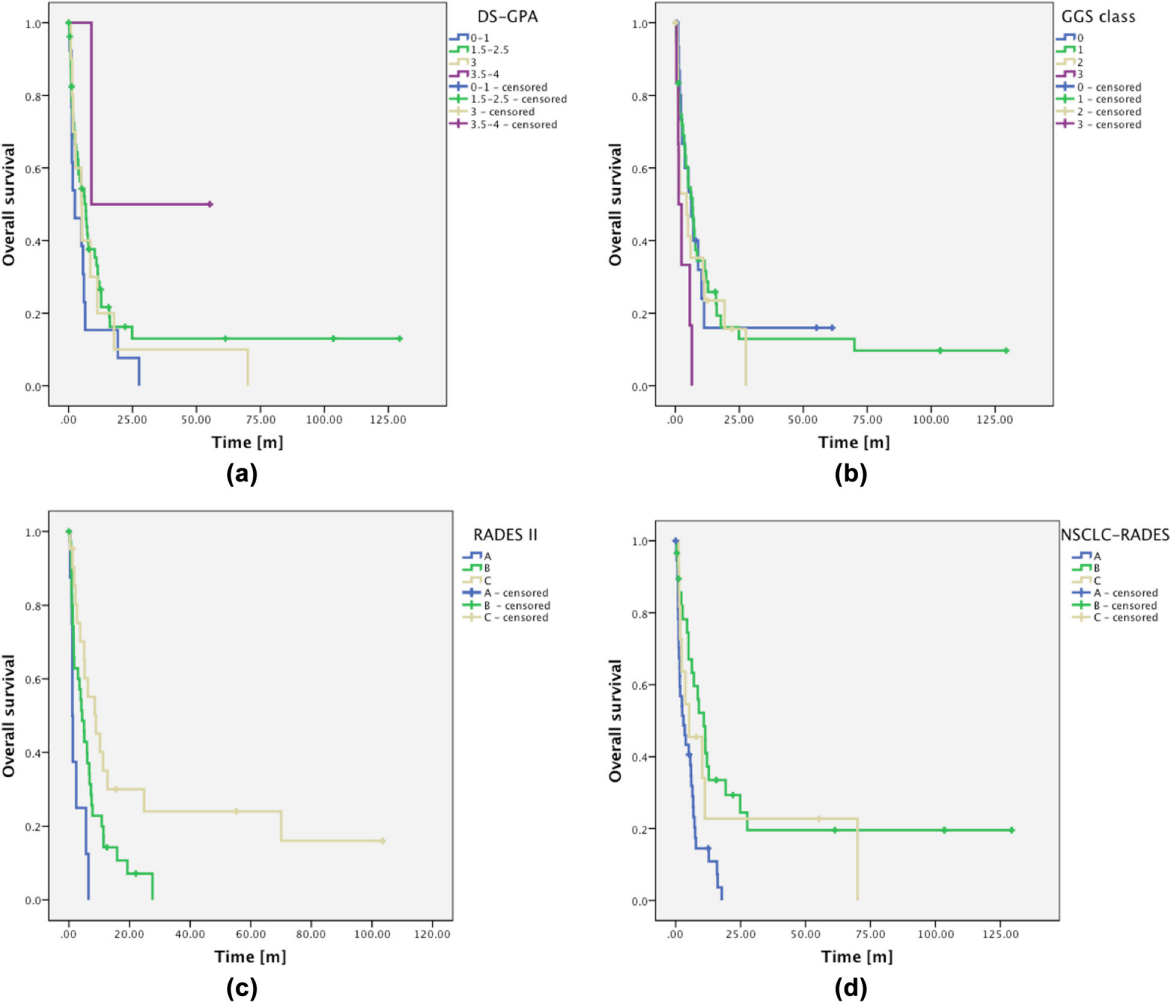
Kaplan-Meier analysis of OS rates grouped according to DS-GPA (**a**), GGS (**b**), RADES II (**c**) and NSCLC-RADES (**d**). Note that group b shows a shorter median overall survival rate than group c

Several factors have led to an increase in the incidence of BM including an aging population, improvements in imaging techniques as well as systemic therapies that do not effectively penetrate the blood–brain barrier [4]. Morbidities caused by BM include neurologic deficits and cognitive decline and around 20 % of cancer deaths are linked to intracranial metastases.

Overall prognosis for patients suffering from BM is generally poor with a median overall survival (OS) of less than 1 year. However, OS may vary significantly between different patients depending on prognostic factors such as performance status, extracranial disease status, number of metastatic lesions, tumor size, and histology among others [4]. Several studies have thus tried to identify prognostic markers in order to identify subgroups of patients that are more likely to benefit from aggressive therapy [5, 6].

Traditionally BM were managed by whole brain radiotherapy (WBRT), which has been shown to improve survival as well as quality of life (QOL) [7]. Points of criticism of WBRT are treatment times of 2–3 weeks, preclusion of concurrent chemotherapy as well as a decline in neurocognitive function in long-term survivors [8]. As a consequence, several studies have evaluated the role of local approaches such as surgery or stereotactic radiosurgery (SRS), either alone or in combination with WBRT [9]. Some studies have shown that stand-alone local therapies minimize neurocognitive long-term impairment and improve QOL without compromising OS [10]. Other studies have shown higher rates of intracranial relapse but not worse survival rates in patients who received SRS compared to patients who received a combination of SRS and WBRT [11]. In everyday clinical practice, WBRT is commonly used for palliative patients, while local therapies are reserved for patients with longer life expectancy.

Several authors have tried to develop prognostic indices in order to facilitate decision making when treating BM patients. Four recently published indices are the Golden Grading System (GGS), the disease-specific graded prognostic index (DS-GPA), the second prognostic index published by Rades et al. in 2011 (RADES II) as well as the NSCLC-specific index published by RADES et al. in 2013 (NSCLC-RADES) [12–15]. These indices are helpful when discussing treatment decisions of NSCLC BM patients. While the GGS uses the factors age, Karnofsky Performance Score (KPS) and presence of extracranial metastases (ECM), the NSCLC DS-GPA includes these three factors as well as the number of cranial metastases. The RADES II also uses age, KPS, ECM and number of BM and adds the interval from primary tumor diagnosis to radiotherapy as a parameter. The NSCLC-RADES is calculated using the factors gender, KPS and ECM. These indices have become accepted in everyday clinical life because most other indices developed to this date use components that are difficult to quantify or are subjective (e.g. control of extracranial disease or BM volume) [6].

It was the aim of this study to evaluate the prognostic value of these four indices for the NSCLC BM patient population seen in our department that received stereotactic radiotherapy as primary BM treatment.

## Methods

### Treatment decisions, patient selection and dose regimens

We performed a retrospective analysis on 90 patients who underwent stereotactic radiosurgery (SRS) or fractionated stereotactic radiotherapy (FSRT) of intracranial NSCLC metastases between 04/2004 and 05/2014 and who had not undergone prior surgery or WBRT for BM. Follow-up data was analyzed until May 2015.

In our institution, treatment decisions are based on an interdisciplinary vote and treatment planning is decided upon individually for every single case. Planning depends not only on the size of the tumor but also on the location and KPS. High single doses were considered SRS and doses applied in multiple fractions were considered FSRT. Generally speaking, small tumors with a volume ≤10 ccm or tumors distant to critical structures were treated with SRS, while larger tumors >10 ccm or tumors in close proximity to critical structures were assigned to FSRT. However, there could be exceptions made for individual cases.

### Stratification and variables

Patients were stratified according to age, gender, KPS, histology, International Union Against Cancer (UICC) stage, number of treated lesions, prior surgery on the primary tumor, total planning target volume (PTV), highest biologically effective dose (BED_10_) per patient, synchronous vs. metachronous diagnosis of BM (>1 month after NSCLC diagnosis was considered metachronous), tumor localization, presence of ECM and interval from BM diagnosis to radiotherapy. The four prognostic scores DS-GPA, RADES II, NSCLC-RADES and GGS were calculated.

Salvage therapy after the first radiotherapy was noted as well. Patients with tumor progress could undergo salvage SRS, WBRT or a resection of the BM.

Follow-up examinations, including MRI as well as clinical and neurologic examinations were performed at 6–8 week intervals after radiotherapy.

### Technical set-up

Patients were treated using Novalis^®^ (BrainLab^®^) with beam shaping capability, built-in multi-leaf collimator (MLC) and image guidance. The Novalis ExacTrac^®^ image guided frameless system enabled us to image the patient in any couch position using a frameless positioning array. Magnetic resonance imaging (MRI)/ computed tomography (CT) fusion planning was performed. The three-dimensional treatment planning system iplanRT^®^ was used. Gross tumor volume (GTV) was defined as the area of contrast enhancement on T1-weighted MRI images, the PTV included a 1–2 mm isotropic safety margin. If fusion images were considered to be of good quality, the PTV margin used was only 1 mm. If fusion images were not considered adequate, a safety margin of 2 mm was used. The dose was prescribed to the 80 % isodose at the PTV margin.

### Formulas and statistics

The BED was calculated for every metastasis treated according to the following formula, where n is the number of fractions and d the dose per fraction. Following the Linear quadratic model, a value of ten was used for the *α*/*β*-ratio.

1 $$ BED=nd\left[1+\frac{d}{\alpha /\beta}\right] $$

OS started with the first day of irradiation and was estimated using Kaplan-Meier analysis. Subgroups were compared using the log-rank test for univariable analysis and the Cox proportional hazard model for multivariable analysis. A p-value of less than 0.05 was considered statistically significant. A p-value of less than 0.1 was considered a trend and was the criterion for inclusion in multivariable analysis. All statistical analyses were performed using IBM SPSS Statistics 19 (New York, USA).

## Results

### Patients

Patient characteristics are summarized in detail in Table 1.

**Table 1:** Characteristics of the 90 BM patients analyzed

Characteristics	No./median (range)	%
Sex (m/f)	57/33	63.3 %/36.7 %
Age (y)	63.3 (38.9–83)	
KPS		
100	6	6.7 %
90	12	13.3 %
80	25	27.8 %
70	21	23.3 %
60	6	6.7 %
50	7	7.8 %
n/a	13	14.4 %
Histology		
Adenocarcinoma	55	61.1 %
Squamous cell carcinoma	19	21.1 %
Large cell carcinoma	6	6.7 %
Other	8	8.9 %
n/a	2	2.2 %
UICC stage at time of first diagnosis		
I	5	5.6 %
II	7	7.8 %
III	7	7.8 %
IV	67	74.4 %
n/a	4	4.4 %
Synchronous BM	42	46.7 %
ECM	51	56.7 %
Number of treated lesions		
1	55	61.1 %
2	24	26.7 %
≥ 3	11	12.2 %
Total BM volume (PTV) per patient (ccm)	2.32 (0.2–45.2)	
Highest BED_10_ per patient	91.14 (20.98–97.36)	
Fractionation		
SRS	108	78.8 %
FSRT	28	20.4 %
n/a	1	0.7 %
Localization		
Temporal lobe	15	10.8 %
Occipital lobe	29	20.9 %
Parietal lobe	34	24.5 %
Frontal lobe	28	20.1 %
Cerebellum	21	15.1 %
Brainstem	5	3.6 %
Basal ganglia	4	2.9 %
n/a	3	2.2 %
Salvage WBRT	13	14.4 %
Salvage SRS or FSRT	10	11.1 %
Salvage resection	5	5.6 %

*n/a* not available

90 patients treated for 137 BM in our department between 04/2004 and 05/2014 were included in the analysis. The majority of the patients were male (63.3 %) and the median age was 63 years. Most Patients (71.1 %) had a good KPS of 70 % or higher. Most patients were already diagnosed UICC stage IV at the time of cancer diagnosis (74.4 %) and almost half of the patients showed synchronous brain metastases (46.7 %). Adenocarcinoma was the most common histologic subtype of cancer (61.1 %).

The majority of patients (56.7 %) also showed ECM. More than half of the patients (61.1 %) were initially treated for a single brain metastasis. Median total lesion volume was 2.32 ccm.

Metastases were treated with SRS (78.8 %) or FSRT (20.4 %). SRS was usually administered with 25.6 Gray (Gy) (90.7 %). FSRT was mostly conducted in 11 or 13 fractions (64.3 %) and a single dose of 3.8 Gy, resulting in a total dose of 41.8 or 49.4 Gy. The median of the highest BED_10_ per patient was 91.14 Gy (range 20.98–97.36 Gy).

### Overall survival

Of patients alive at last follow-up, median follow-up time was 14 months. The OS rate was 51 % after 6 months and 29.9 % after 12 months. After 2 and 5 years, 15.7 and 9.1 % of the patients were still alive, respectively.

The univariable analysis of potential predictive factors is shown in Table 2. Statistically significant factors of better OS were lower UICC stage at first diagnosis, adenocarcinoma histology, lower total BM volume and prior surgery on the primary tumor. There was a trend towards better survival rates for lower KPS, metachronous BM and absence of ECM.

**Table 2:** Univariable analysis of potential preditive factors

Log-Rank test	
Variable	*p*
Age (< vs. ≥ median)	0.65
Sex	0.42
KPS (< vs. ≥ median)	0.09 (**)
UICC stage	0.020 (*)
Histology (adeno vs. other)	0.016*
Prior surgery	0.001 (*)
Single vs. multiple lesions	0.41
Number of lesions	0.59
Synchronous BM	0.065 (**)
ECM	0.076 (**)
Total BM volume (PTV) per patient,(< vs. ≥ 5ccm)	0.026 (*)
SRS vs. FSRT	0.84
Highest BED_10_ per patient (< vs. ≥ median)	0.52
Interval PT diagnosis to RT (< vs. ≥ median)	0.17

* *p*-value ≤ 0.05; ** *p*-value ≤ 0.1

In multivariable analysis higher KPS, adenocarcinoma histology and presence of ECM were significant predictive factors (Table 3).

**Table 3:** Multivariable analysis of potential preditive factors

Variable	HR	95 % CI	*p*
KPS (in %, continuous)			0.042 (*)
50 %	7.16	1.61–31.73	
60 %	2.52	0.59 - 10.80	
70 %	1.09	0.29–4.12	
80 %	1.84	0.53–6.45	
90 %	1.81	0.46–7.13	
100 % (reference)			
Histology (adeno vs. other)	0.34	0.17–0.67	0.002 (*)
ECM	2.003	1.09–3.69	0.026 (*)

The analysis included all factors with *p*-values ≤ 0.1 in the log-rank test.

* *p*-value  ≤ 0.05

### Prognostic indices

An overview of the prognostic value of the four examined indices is given in Fig. 1 and Table 4. In our patient population the RADES II and the NSCLC-RADES index were significant predictors of OS in univariable analysis, while the DS-GPA and the GGS were not statistically significant. However, the NSCLC-RADES failed to differentiate between intermediate- and low-risk patients: Intermediate-risk patients according to the NSCLC-RADES showed far better median OS rates than low-risk patients (10.8 months vs. 5.2 months). High-risk patients had the worst OS rates (3 months).

**Table 4:** Univariate analysis of prognostic scores

	Log-Rank test *p*-value
Variable	OS
GGS	0.11
DS-GPA	0.22
RADES II	0.001 (*)
NSCLC-RADES	0.002 (*)

*p-value ≤ 0.05

## Discussion

The question which BM subpopulation benefits from brain radiotherapy has been a controversial issue [16]. Prognostic indices may be used to identify subpopulations eligible for more intensive therapies. In this study we retrospectively examined four of the more recently published prognostic indices in a patient population treated with SRS or FSRT in our department. These indices are simple to calculate and do not rely on subjective variables.

Several studies have tried to compare established prognostic indices for BM patients. In a recent systematic review by Rodrigues et al. from 2013 no index could be identified as superior [17].

All examined indices (GGS, DS-GPA, RADES II and NSCLC-RADES) use the KPS and the presence of ECM. Both variables were highly significant predictors after multivariable analysis in our patient collective.

The GGS, DS-GPA and RADES II use the factor age, which was not a significant predictor either in univariable or in multivariable analysis. Some authors have questioned the predictive value of the factor age in NSCLC populations and even the authors of the NSCLC-RADES study did not find age to be a significant predictor in a homogenous NSCLC population either [15].

The RADES II as well as the DS-GPA also use the number of BM, which was not a predictive factor in our patient collective either. However, the total volume of BM was highly predictive in univariable analysis. The first study of prognostic scores for patients in whom SRS alone was used to treat newly diagnosed BM was published by Likhacheva et al. in 2013 [18]. In their patient collective, BM volume was a predictive factor for OS in multivariable analysis, while the number of BM was not a significant factor [18]. The authors argue that the decision whether to use stereotactic radiotherapy or WBRT should not be based on the number of BM a patient presents, but rather on BM volume.

The interval from diagnosis to radiotherapy is only used in the RADES II, we did however not find it to be significant either in univariable or in multivariable analysis.

The NSCLC-RADES is the only examined index including the factor gender, which was not predictive in our study.

The factor we found to be the most significant is not used in any of the examined indices: Adenocarcinoma histology of the tumor. It must be noted that none of the studies that led to the definition of the indices examined here did analyze histological subtypes of NSCLC. This result is also in accordance with data published by Kuremsky et al. who showed significant better OS rates for adenocarcinoma compared to squamous cell carcinoma (SCC) [19]. A possible explanation for this phenomenon is the fact that SCC is often diagnosed at a higher stage than adenocarcinoma. A second reason might be the availability of newer systemic agents for adenocarcinoma patients (e.g. Pemetrexed and Erlotinib).

To substantiate this possible explanation Kuremsky et al. compared patients from the pre-Pemetrexed and -Erlotinib era (before 2005) with patients treated after 2005, however the authors did not find significant differences.

When comparing our data with the original works by Sperduto, Rades and Golden, it must be kept in mind that the patient population examined here is much more homogeneous in terms of treatment than in the original works, since we excluded all patients who had received WBRT or BM resection in the first place. The collective analyzed in the NSCLC-RADES study was treated with WBRT only, while we only included FSRT and SRS patients.

This may to some extent explain the differences in statistical significance of the examined factors.

The fact that the RADES II and the NSCLC-RADES gave better results may in part be explained by the fact that patients are grouped in three classes instead of four classes as in the DS-GPA and GGS. Furthermore, the RADES II and the NSCLC-RADES place more weight on the KPS and the presence of ECM than the GGS and the DS-GPA, which appeared to be prognostic factors in our study as well and thus may have contributed to the improved results for these two scores.

Finally, the limitations of this study should be mentioned. Firstly, the retrospective nature of the analysis is prone to bias. Secondly, the number of patients may have been too low to find significance of some potential prognostic factors (e.g. Interval from tumor diagnosis to radiotherapy) or even of the indices GGS and DS-GPA themselves.

## Conclusion

Multiple prognostic indices for BM patients have been developed and are currently in use, but the ideal index has not been defined yet and further research into alternative approaches is needed. Of the indices examined here, the RADES-II showed the best results for a SRS and FSRT-treated patient collective with NSCLC BM. Our results indicate that the factor adenocarcinoma histology could add to the prognostic value of specialized future indices.
